# On the Publishing of an Annual List of All the Regular Medical Practitioners in the Kingdom

**Published:** 1801-10

**Authors:** 

**Affiliations:** Scotland


### *On the publishing of an Annual List of all the regular Medical Practitioners in the Kingdom.*

To the Editors of the Medical and Physical Journal,

Gentlemen,

On the importance of the Medical Profeffion it is not neceffary to dwell; all are interefted in it, for all have health to preferve or to regain. The efteem in which the guardians of health have been held in all ages, is a practical proof of the utility and importance of medicine. The majority of the profeffion muft have excelled other claffes of the community, in refpect both of information and ufefulnefs, before they could have attained their prefent degree of diftinction. Refpectability will not, with general confent, be long mifplaced.

There

There will, however, always be found men bafe enough to make pretenfions though deftitute of merit, and to claim refpect and confidence by affuming the badge of the profeffion. The intereft of the public unites with that of the medical profeffion in requiring as much purity and merit as poffible, from a body of men entrufted with what is moft valuable to mankind.

To the Faculty we muft leave the right of afcertaining the capability of perfons for practifing medicine, as they are the fitteft to decide. But it is furely not improper that the public be informed of thofe who are accounted qualified by fuch authority, and that the life of any member of the community be not committed to every EMPIRIC who has the impudence to practife, or to every *impostor* who has the audacity to place his name on a fign-board, with *Surgeon* or *Physician* falfely attached to it as an *appendage*.

In Scotland not above two-thirds of thofe who call themfelves phyficians and furgeons, have ever obtained *degrees* or *diplomas*. It is not uncommon for men, deftitute of a liberal education, to attend a drug-fhop for a few months, the medical claffes for a feffion, and forthwith find themfelves *qualified* to commence phyficians or furgeons! There are even numbers practifing as phyficians and furgeons who never attended a medical clafs!

The felf-conceit that appears in the half-inftructed of every profeffion has long been obferved, and we need not be furprifed at the extravagance of human pretenfions. The fcience of medicine is fo clearly detailed in books, and the treatment of difeafes fo diftinct, that to one ignorant of practice, but acquainted with Treatifes on Medicine, every complaint is of eafy cure. This may be partly illuftrated by an anecdote: A fhrewd obferver of human nature, faid to a young man entering upon the ftudy of medicine, "You are going to the Univerfity, and after the firft year you will think you can cure every difeafe; after the fecond, you will find many complaints to which you can do nothing; after the third, you will fuppofe that you can cure almoft none." It requires much ftudy to know our own ignorance; the danger of men who have only a fmattering of medical knowledge may be eafily conceived, and is frequently felt.

I was led to thefe reflections by the following circumftance. A friend of mine, in a tour through the weft of England, unfortunately fell from his carriage and broke one of his thigh bones. A furgeon, or at leaft a perfon who had affumed the name, was inftantly called to fet the bone. This fellow, completely ignorant of the proper method of treating a fracture, left the knee-joint perfectly extended, inftead of placing the limb limb in a relaxed pofition. The confequence was, that the mufcles being put to the ftretch, one end of the bone paffed the other near two inches. The patient indeed recovered, after being confined to a couch more than fix months; but to his furprize, found that one limb was fhorter than the other by two inches. What an irretrievable misfortune, owing to an ignorant pretender interfering!

This fellow I afterwards underftood, had attended a drug-fhop for a few months, then commenced for himfelf in that line; but finding he did not fucceed, he immediately removed to a part of the country where he was not known, and commenced furgeon at once; and, unfortunately, my friend happened to be among the firft of his patients, or, I may rather fay, victims. In this inftance only a limb fuffered; but in how many cafes has fuch ignorance coft men their lives?

A man of this defcription can have no excufe; he muft be confcious of his own ignorance, and therefore, in many inftances, he muft be running the rifk of injuring the health of his patients, or perhaps of depriving them of their lives. Men, who have no other feelings than thefe, who have no other fenfe of right and wrong, ought to be deprived of their power of injuring. Better for the world that a thoufand of fuch wretches perifh, than that one unfortunate victim fhould lofe his life by their ignorance and prefumption.

May not the public exercife a kind of cenforfhip in this cafe, fince the profeffion have not taken any vigorous meafures to prevent it? And may not their exertions roufe from flumber thofe whofe authority has been long dormant? We are informed of the members and managers of different corporate bodies in Scotland through the medium of Almanacks; this anfwers different important purpofes: The fame method might be employed in the cafe of phyficians and furgeons; information might be eafily communicated in this manner, the public warned againft impoftors, and the ignorant checked in their infolence. The public cannot be thought adequate judges of medical fkill, but they certainly deferve to be told who are the perfons properly qualified to exercife that profeffion.

This defirable end, in my opinion, might be very eafily accomplifhed. Let the Editor of fome refpectable Almanack publifh a few advertifements in the different news-papers, fignifying his intention to publifh, in his Almanack, a complete lift of all the phyficians and furgeons in Scotland, who have obtained regular degrees and diplomas; I am convinced that every one poffeffed of thefe qualifications would fend him a certificate of the fame, free of all expence. The only difficulty that would occur is in making out the firft lift; after that is accomplifhed accomplifhed it would become a regular bufinefs. Every ftudent, as foon as he had obtained his degree or diploma, and had determined in what town he meant to fettle, would find it his intereft to have his name immediately inferted in the Almanack, to prevent his being ranked among quacks and impoftors. And, in order to make this lift as public as poffible, I would advife, that for a year or two at firft, it be publifhed not only in the almanacks, but alfo in the different news-papers and magazines in the kingdom. By thefe means every man might be informed of the regular practitioners in his part of the country; and if he afterwards employed an impoftor, he had himfelf to blame.

The lift ought to contain, firft, the name of the county; fecond, the name of the town; third, the name of the practitioner; and, fourth, the place and date of the degree or diploma. By adding the place and date of the degree or diploma, an effectual check would be given to perfons who might wifh to foift their names into the lift without being properly qualified; for it would be the intereft of the different Faculties to take care that none entered this lift who had not received their fanction.

This notification is not only proper to the inhabitants of diftricts where medical men refide, but alfo ufeful to ftrangers who may occafionally require their affiftance when travelling through the country. The influence of this meafure on medical men would not be fmall; the preparations neceffary, before a ftudent can offer himfelf to the examination of a faculty, requires refearch: In the courfe of inquiry, knowledge is acquired; many fubjects come under review, and prefent themfelves to attention, which, otherwife, would not have been confidered, till, in the hurry of practice, a puzzling cafe had forced them upon the notice of the practitioner; and it is, furely, better to acquire previous information, than to obtain knowledge at the expence of the health or life of patients.

Man is apt to be indolent, but the profpect of a public examination, preparatory to receiving a degree or diploma, is no fmall ftimulus to diligence. Inftead of general and incorrect notions upon the fymptoms, the caufes and cure of difeafes, the phyfician muft defcend into an accurate inveftigation of the nature of the complaint, and acquire a knowledge of remedies, and of treatment adapted to particular diforders; while the furgeon muft obtain minute acquaintance with the ftructure of the human frame, with its form in a found ftate, its appearance when injury has been received, and the moft approved methods of performing the different operations of furgery. Their information muft be accurate, for they are to be examined by men of experience, and approved or rejected in proportion as they have acquired useful knowledge, or misimproved their opportunities.

It is certain, that one-fourth part of the students who apply for degrees and diplomas at the Faculties of Edinburgh and Glasgow, are rejected as unqualified. This proves, that students are apt to rate their acquisition of knowledge too high, and think themselves qualified, when, in fact, they are mere novices. Time is requisite for acquiring knowledge; and he who bestows attendance, is in the way of picking up useful information. We are, at least, assured, when a degree or diploma has been procured, that the usual attendance has been given, and that the usual examination has taken place. We can partly be acquainted with the nature of such examination, and value of the degree or diploma, if we know whence they were received.

There has been a general and just outcry against *quack medicines*; their baneful effects have been deeply felt by every part of the community. This measure, were it once brought to maturity, would have a tendency to lessen the number and estimation of *quack practitioners*, whose individual injury, though not so extensive as the former, do often more prejudice in those parts of the country in which they reside.

It is a piece of justice we owe to those who are legally qualified, it is a duty which we owe to the public, it is the interest of medical societies and faculties to promote its influence, and it recommends itself to those who publish these vehicles of intelligence. The publisher, who shall first give a correct list of all the medical gentlemen in Scotland, deserves their approbation and encouragement, as well as the approbation and encouragement of all the friends of suffering humanity. It might be useful too, in ascertaining an important question, "If the medical profession be attended with greater mortality than others?" It would certainly contribute to preserve the purity and advance the respectability of an honourable profession; it would aid the views of philanthropy, and promote the interest of science.

I am, &c.

*Scotland, Sept.* 10, 1801. PHILO-MEDICUS.

